# Diagnostic Performance of Shear-Wave Elastography in Autoimmune Hepatitis: Evaluating Baseline Characteristics and Accuracy Across Fibrosis Stages

**DOI:** 10.7759/cureus.76789

**Published:** 2025-01-02

**Authors:** Nida Rasool, Ali Hyder, Khaild Tareen, Imran Ahmed, Samra Iqbal, Marium Kauser Siddiqui, Raja Taha Yaseen Khan, Nasir Hassan Luck

**Affiliations:** 1 Hepatogastroenterology, Sindh Institute of Urology and Transplantation, Karachi, PAK; 2 Gastroenterology, Chandka Medical College, Shaheed Mohtarma Benazir Bhutto Medical University, Larkana, PAK; 3 Gastroenterology, Shaikh Khalifa Bin Zayed Al Nahyan Hospital, Quetta, PAK; 4 General Internal Medicine, Princess Royal University Hospital, King’s College Hospital NHS Foundation Trust, London, GBR; 5 Medicine, Civil Hospital Karachi, Karachi, PAK; 6 Medicine and Surgery, Dow University of Health Sciences, Civil Hospital Karachi, Karachi, PAK; 7 Gastroenterology, Sindh Institute of Urology and Transplantation, Karachi, PAK

**Keywords:** autoimmune hepatitis (aih), diagnostic accuracy, liver fibrosis, non-invasive, shear-wave elastography (swe)

## Abstract

Background

Autoimmune hepatitis (AIH) is a chronic inflammatory condition affecting the liver, ultimately leading to fibrosis, followed by cirrhosis and liver failure. Although liver biopsy is necessary to confirm the disease and outline different stages of liver fibrosis, it is associated with several risks due to its invasiveness. Shear-wave elastography (SWE) has recently emerged as a non-invasive imaging modality, representing liver stiffness and providing additional fibrosis assessment. This study aims to validate SWE for diagnosing and discriminating liver fibrosis stage in AIH patients.

Methodology

This retrospective cohort study was conducted at the Sindh Institute of Urology and Transplantation, Karachi, with the enrollment of 162 patients diagnosed with AIH between March 2022 and December 2023. All participants underwent SWE and liver biopsy. The stages of fibrosis were assessed using the Metavir scoring system. Demographic, biochemical, and serological data were analyzed, including autoantibody and serum IgG levels. Diagnostic accuracy, sensitivity, and specificity of SWE were compared with histopathological findings.

Results

Of the 162 patients, 71 (43.8%) were males, and 91 (56.2%) were females, with a mean age of 35.8 ± 16.6 years. SWE had an overall diagnostic accuracy of 92.11%, sensitivity of 82.86%, and specificity of 98.7% in predicting fibrosis stages. SWE was effective in differentiating early fibrosis (F1-F2), while it showed reduced sensitivity for advanced fibrosis (F3-F4). Female gender (p = 0.009), elevated bilirubin (p = 0.009), autoantibody titers, and serum IgG levels (p = 0.043) were significant factors related to advanced fibrosis.

Conclusions

SWE has shown high diagnostic accuracy and specificity for the estimation of liver fibrosis in AIH, especially in the early stages. Sensitivity is lowered in advanced fibrosis. SWE is a very valuable non-invasive alternative to liver biopsy. Its use may decrease procedural risks, accelerate diagnosis, and improve outcomes in AIH. Further multicenter studies are recommended for confirmation and to explore the comparative performance of SWE against other non-invasive approaches.

## Introduction

Autoimmune hepatitis (AIH) is an immune-mediated inflammatory process that progressively leads to chronic liver disease, decompensation, and liver failure, ultimately requiring liver transplantation [1]. Its onset has a bimodal distribution, with age peaking at 10-30 years and 40-60 years, predominantly occurring in the female population (71-95% women) [2]. The annual incidence of AIH ranges from 0.67 to 2.0 cases per 100,000 depending on the geographical distribution [3]. The incidence has increased over the years, and its clinical presentation varies in different ethnic groups, with Asians and other non-European individuals having poor disease outcomes.

Early diagnosis is crucial as untreated AIH has a high mortality rate. Around one-third of adult patients and a half of children with AIH have cirrhosis at the time of presentation [4]. The diagnosis of AIH is based on histological, clinical, and laboratory findings (elevated serum aspartate aminotransferase (AST) and alanine aminotransferase (ALT) levels and increased serum immunoglobulin G (IgG) concentration) and the presence of autoantibodies specific for AIH [5]. Histopathological findings include lymphoplasmacytic infiltrates and the presence of rosettes, emperipolesis, and plasma cells, affecting the interface, commonly described as interface hepatitis [3].

The aim of treatment in AIH is to achieve disease remission and prevent further progression of liver disease, achieved with the administration of steroids and immunosuppressant therapy [4]. Liver fibrosis results in the development of portal hypertension, including ascites, variceal hemorrhage, hepatic encephalopathy, as well as hepatocellular carcinoma [5].

There are invasive and non-invasive methods for diagnosing and monitoring disease progression. Although liver biopsy is the gold standard for diagnosing fibrosis and follow-up treatment response, it is associated with significant risk and potential complications of pain, bleeding, infection, perforation, and even death [6]. Mehran et al. reported that post-biopsy hemorrhage is the major cause of mortality, occurring in up to 10.9% of cases [6]. In contrast to liver biopsy, electrography is accessible and repeatable, making it easier to use at a population level to evaluate fibrosis.

Furthermore, liver biopsy has a few limitations due to its small specimen size, specimen mishandling, inter and intraobserver reliability with histologic assessment, and the associated risk of bleeding and infection [7]. Hence, there is a need for a non-invasive and accurate method to characterize fibrosis in individuals with AIH.

Elastography helps in the estimation of liver fibrosis. Two-dimensional shear-wave elastography (2D-SWE) is a real-time two-dimensional elastography technique that relies on acoustic radiation force to generate shear waves, determining liver stiffness quantitatively [8-10]. Elastography has a well-established role in the staging of liver fibrosis in patients with hepatitis B-related chronic liver disease [11]. However, limited studies are available regarding the role of elastography in AIH. According to previous studies, serum tests are inferior to imaging tests when evaluating fibrosis in AIH and overlap diseases [12].

Determination of a non-invasive index of AIH will be helpful for the community in predicting fibrosis and disease severity without the need to perform a liver biopsy and identify individuals at risk of future complications such as liver cirrhosis. Therefore, in this study, we aimed to determine the diagnostic performance of SWE by comparing histologic liver fibrosis with SWE measurements of liver stiffness.

## Materials and methods

After obtaining approval from the Ethical Review Committee, Sindh Institute of Urology and Transplantation (approval number: 839), this retrospective cohort study was conducted at the Hepatogastroenterology Unit of the Sindh Institute of Urology and Transplantation, Karachi. Informed consent was obtained from all participants who met the inclusion criteria. All patients diagnosed with AIH as per the operational definition who had received a liver biopsy and undergone SWE from March 2022 to December 2023 were enrolled in the study (Table 1). Patients with concomitant liver diseases and those with missing data were excluded from the study.

**Table 1:** Operational definitions of AIH, SWE, and liver biopsy. AIH = autoimmune hepatitis; 2D-SWE = two-dimensional-shear-wave elastography

Operational definitions
AIH [4]: Patients are diagnosed with AIH if there is at least one elevation of an aminotransferase, typically at least two times the upper limit of the normal range, as well as elevation of an associated autoantibody (e.g., anti-smooth muscle antibody, antinuclear antibody, anti-liver-kidney microsomal-1 antibody), presence of raised IgG levels, and biopsy finding compatible with AIH
2D-SWE [8]: Measurements were performed by an experienced radiologist. All patients were asked to fast at least four hours before the examination and hold their breath in the supine position when 2D-SWE was performed. The 2D-SWE box was placed 1.5–2 cm under Glisson’s capsule of the liver and in the parenchyma area of the right hepatic lobe that was free of large bile ducts and vessels. A few consecutive SWE images were obtained for each patient and the average and median of these measurements expressed in kPa were used to estimate the degree of liver stiffness for each subject and compared with the biopsy Metavir score. SWE cutoff for different stages of liver fibrosis: F1 <7.2 kPa, F2 ≥7.2 kPa, F3 ≥13.2 kPa, and F4 ≥ 16.3 kPa
Liver biopsy [8]: Liver fibrosis was evaluated using the Metavir scoring system. Liver fibrosis was staged as follows: Stage 0: absence of fibrosis, Stage 1: portal fibrosis without septa, Stage 2: peri-portal or portal-portal septa, Stage 3: fibrous septa with architectural distortion, and Stage 4: cirrhosis

Data collection

Demographic data including baseline laboratory investigations such as complete blood count; liver function tests; serological tests including autoimmune antibodies such as antinuclear antibody (ANA), anti-smooth muscle antibody (ASMA), and anti-liver-kidney microsomal antibody (anti-LKM); and serum IgG levels were recorded for each patient. Liver fibrosis was evaluated using the Metavir scoring system on liver biopsy by a histopathologist with more than 20 years of experience in hepatobiliary pathology. SWE measurements were performed by an experienced radiologist with more than five years of experience in performing the procedure, with patients fasting for at least four hours before the examination. SWE results were compared with histologic findings from liver biopsies.

Data analysis

The data were entered and analyzed using SPSS version 25.0 (IBM Corp., Armonk, NY, USA). Continuous variables are expressed as mean ± standard deviation, while categorical variables are expressed as frequencies and percentages. The outcome was measured in terms of the presence or absence of advanced liver fibrosis on histopathology. The chi-square test was utilized for the comparative analysis of categorical variables while continuous variables were compared using the Student’s t-test. Lastly, the diagnostic accuracy of SWE was obtained in predicting mild, moderate, and severe fibrosis and cirrhosis.

## Results

A total of 162 patients were enrolled in the study, comprising 71 (43.8%) males and 91 (56.2%) females. The mean age of the participants was 35.8 ± 16.6 years. Baseline characteristics are presented in Table 2. Hematological parameters revealed a mean hemoglobin level of 10.5 ± 1.65 g/dL, a total leukocyte count (TLC) of 6.3 ± 3.9 × 10⁹/L, and a mean platelet (PLT) count of 177 ± 146.2 × 10⁹/L. Biochemical analyses showed a mean total bilirubin level of 4.0 ± 6.3 mg/dL, alkaline phosphatase of 191 ± 170.4 IU/L, AST of 115.9 ± 188.9 IU/L, ALT of 83 ± 115.2 IU/L, and Gamma-glutamyl transferase (GGT) of 135.8 IU/L (±156.1). The mean serum albumin was 3.6 ± 0.75 g/L, with an international normalized ratio (INR) of 1.1 ± 0.26.

**Table 2:** Baseline characteristics of the study population (n = 162). ANA = antinuclear antibody; ASMA = anti-smooth muscle antibody; LKM = liver-kidney microsomal; TLC = total leukocyte count; PLT = platelet; AST = aspartate transaminase; ALT = alanine transamianse; GGT = gamma-glutamyl transferase; INR = international normalized ratio; IgG = immunoglobulin G

Variable		n (%)
Gender	Male	71 (43.8)
	Female	91 (56.2)
ANA (titers)	1:80	26 (16)
	1:160	97 (59.9)
ASMA		47 (29)
Anti-LKM antibody		4 (2.5)
Negative serology		23 (14.2)
Serum IgG (mg/mL)		22.9 ± 8.1
Shear-wave elastography	F1 <7.2 kPa	50 (30.9)
	F2 ≥7.2 kPa	44 (27.2)
	F3 ≥13.2 kPa	40 (24.7)
	F4 ≥16.3 kPa	28 (17.3)
Liver fibrosis on histopathology	Mild fibrosis	49 (30.2)
	Moderate fibrosis	43 (26.5)
	Severe fibrosis	40 (24.7)
	Cirrhosis	30 (18.5)
Age (years)		35.8 ± 16.6
Hemoglobin (g/dL)		10.5 ± 1.65
TLC (×10⁹/L)		6.3 ± 3.9
PLT (×10⁹/L)		177 ± 146
Total bilirubin (mg/dL)		4.0 ± 6.3
Alkaline phosphatase (IU/L)		191 ± 170.4
AST (IU/L)		115.9 ± 188.9
ALT (IU/L)		83 ± 115.2
GGT (IU/L)		135.8 ± 156.1
Serum albumin (g/L)		3.6 ± 0.75
INR		1.1 ± 0.26

On serology, 26 (16%) patients had ANA titers of 1:80, while 97 (59.9%) patients had ANA titers of 1:160. ASMA positivity was observed in 47 (29%) patients, and anti-LKM antibodies were detected in 0.2% of participants. Serology was negative in 23 (14.2%) patients. The mean serum IgG levels were 22.9 ± 8.1 g/L. On SWE, 50 (30.9%) patients had F1 fibrosis, 44 (27.2%) had F2, 40 (24.7%) had F3, and 28 (17.3%) had F4 fibrosis. Liver biopsy results showed mild fibrosis in 49 (30.2%) patients, moderate fibrosis in 43 (26.5%), severe fibrosis in 40 (24.7%), and cirrhosis in 30 (18.5%) patients, respectively.

On comparative analysis, female gender (p = 0.006), raised serum bilirubin levels (p = 0.009), alkaline phosphatase (p = 0.034), ALT (p = 0.021), AST (p = 0.034), INR (p ≤ 0.001), decreased TLC (p ≤ 0.001) and PLT (p ≤ 0.001), higher ANA titers (p = 0.001), ASMA positivity (p = 0.005), and raised serum IgG levels (p = 0.043) were significantly associated with the presence of advanced liver fibrosis on histopathology. SWE also showed a clear progression of fibrosis, with F1 and F2 stages predominantly seen in patients with mild-to-moderate fibrosis, while F3 and F4 stages were only observed in patients with advanced fibrosis or cirrhosis (p ≤ 0.001) (Table 3).

**Table 3:** Comparative analysis of baseline characteristics regarding the presence or absence of advanced liver fibrosis or cirrhosis (n = 162). ANA = antinuclear antibody; ASMA = anti-smooth muscle antibody; SWE = shear-wave elastography; TLC = total leukocyte count; PLT = platelet; AST = aspartate transaminase; ALT = alanine transamianse; GGT = gamma-glutamyl transferase; INR = international normalized ratio; IgG = immunoglobulin G

Variable		Mild-to-moderate fibrosis (N = 92)	Severe fibrosis or cirrhosis (N = 70)	P-value
Gender	Male	49 (53.3)	22 (31.4)	0.006
	Female	43 (46.7)	48 (68.6)	
ANA (titers)	1:80	8 (8.7)	18 (20)	0.001
	1:160	59 (64.1)	38 (54.2)	
	Negative	25 (27.2)	14 (25.8)	
ASMA	Positive	23 (33.7)	24 (40)	0.005
	Negative	61(66.3)	42 (60)	
SWE	F1 <7.2 kPa	50 (54.3)	0 (0)	≤0.001
	F2 ≥7.2 kPa	32 (45.7)	12 (17.2)	
	F3 ≥13.2 kPa	0 (0)	40 (57.1)	
	F4 ≥16.3 kPa	0 (0)	18 (25.7)	
Age (years)		34.4 ± 16.7	37.6 ± 16.4	0.214
Hemoglobin (g/dL)		10.4 ± 1.8	10.7 ± 1.4	0.356
TLC (×10⁹/L)		7.3 ± 4.7	5.1 ± 2.1	≤0.001
PLT (×10⁹/L)		224 ± 179	116.6 ± 35.6	≤0.001
Total bilirubin (mg/dL)		2.6 ± 5.1	5.2 ± 6.9	0.009
Alkaline phosphatase (IU/L)		155 ± 71	220 ± 214	0.015
AST (IU/L)		105 ± 176	129 ± 203	0.034
ALT (IU/L)		57.6 ± 85.5	89.9 ± 145	0.021
GGT (IU/L)		114.6 ± 116.9	162.9 ± 192.7	0.052
Serum albumin (g/L)		3.7 ± 0.86	3.6 ± 0.64	0.238
INR		1.0 ± 0.172	1.2 ± 0.35	≤ 0.001
Serum IgG (g/L)		21.8 ± 8.9	24.4 ± 6.9	0.043

Overall, SWE had an excellent diagnostic accuracy of 92.11 % in predicting liver fibrosis in patients with AIH with a good sensitivity of 82.86%, an excellent specificity of 98.7%, and a positive predictive value of 97.4%. However, the sensitivity of SWE decreased with the advancement in fibrosis stages from F1 to F4 with an excellent sensitivity of 89.8% in detecting F1 fibrosis to 60% in F4 fibrosis. The specificity and diagnostic accuracy of SWE were high in predicting all stages (F1-F4) of liver fibrosis (Table 4).

**Table 4:** Diagnostic accuracy of SWE in predicting the degree of liver fibrosis. SWE = shear-wave elastography; PPV = positive predictive value; NPV: negative predictive value

SWE	Sensitivity (%)	Specificity (%)	PPV (%)	NPV (%)	Diagnostic accuracy (%)
F1	89.8	94.69	88	95.54	93.21
F2	72.09	89.08	70.45	89.83	84.57
F3	70	90	70	90.16	85.19
F4	60	92	64.29	91.04	86.42
Overall	82.86	98.7	97.4	87.25	92.11

## Discussion

This study assessed the diagnostic performance of SWE in assessing liver fibrosis in patients with AIH. The diagnostic accuracy of SWE was 92.11%, the specificity was 98.7%, and the PPV was 97.4%. However, sensitivity showed a progressive decline with advancing fibrosis stages from 89.8% for F1 to 60% for F4. These findings emphasize the potential of SWE as a non-invasive diagnostic modality and highlight some challenges in the detection of advanced fibrosis.

Our findings are in concordance with previous reports on the efficacy of SWE in assessing liver fibrosis, especially due to chronic hepatitis B and C. Samir et al. reported the high diagnostic accuracy of SWE when staging for fibrosis ≥F2. However, they also documented an overall reduced sensitivity at more advanced, cirrhotic stages, as evident in our findings [10]. Such similarities lead us to believe that the performance of SWE may share some common limitations in different chronic liver diseases regarding the identification of severe fibrosis. Zeng J et al., in their study of autoimmune liver diseases, emphasized the role of SWE in reducing dependence on liver biopsy and observed that the overall accuracy was more than 80% with excellent sensitivity in predicting different stages of liver fibrosis [12]. However, our study predicted a higher diagnostic accuracy of over 90% in predicting advanced liver fibrosis in patients with AIH. The discrepancy may be due to differences in patient groups or study techniques.

Serum biomarkers, including AST and ALT, normally used for assessing fibrosis, have always shown limited sensitivity and specificity, especially in AIH [13,14]. This deficiency underlines the superiority of the imaging modality represented by SWE, which is a quantitative real-time technique for measuring liver stiffness. The strong correlation in this study between SWE-derived values of stiffness and biopsy-confirmed fibrosis stages further validates its diagnostic utility.

The high specificity of SWE, especially for the detection of F1-F2 fibrosis, is indicative of its high efficiency in excluding significant fibrosis among AIH patients, thus preventing these patients from undergoing invasive liver biopsies associated with risks for bleeding, pain, or infection [12]. It also provides a reliable method for staging early fibrosis and can lead to timely intervention that may prevent disease progression.

Our study demonstrated a strong association of advanced liver fibrosis with female gender, increasing age, and high serological markers represented by elevated bilirubin, ANA, and ASMA titer. These results also correspond to the already-accepted epidemiology regarding AIH, where it predominantly presents among females and often has significantly enhanced autoantibody activities [13,14].

High serum IgG levels, typical for AIH, in this study, were significantly associated with severe fibrosis. The correlation of IgG with disease severity is in line with previous studies indicating that IgG may serve as a biomarker for disease severity [15]. The significant association of autoantibody titers with the progression of fibrosis further underlines the diagnostic and prognostic value of serological markers in the management of AIH [16,17].

This study has several limitations. First, the retrospective nature of the study increases the possibility of selection bias and incomplete data, which might impact the findings and observations of this study. Second, the single-center design of this study may limit the general application of these results to a wide and diverse population. Third, although this study established the effectiveness of SWE, it did not make extensive comparisons of SWE with other non-invasive modalities or composite fibrosis scores that might place the modality in its rightful position of relative diagnostic performance.

## Conclusions

This study highlights the diagnostic value of SWE as a non-invasive and reliable method for assessing liver fibrosis in patients with AIH. The high specificity and overall excellent diagnostic accuracy, particularly for early stages of fibrosis, represent an added value with respect to liver biopsy. On the other hand, limitations of sensitivity in advanced fibrosis highlight the need for complementary approaches in severe cases. SWE has the potential to minimize the dependency on invasive procedures, thus improving patient outcomes and streamlining clinical workflows for optimized resource utilization in AIH management.
